# Associations between parental mediation and adolescents' internet addiction: The role of parent–child relationship and adolescents' grades

**DOI:** 10.3389/fpsyg.2022.1061631

**Published:** 2022-12-06

**Authors:** Xiaojing Li, Ying Ding, Xianchun Bai, Lisha Liu

**Affiliations:** ^1^Chinese Academy of Disability Data Science, Nanjing Normal University of Special Education, Nanjing, China; ^2^Bio-X Institutes, Key Laboratory for the Genetics of Development and Neuropsychiatric Disorders (Ministry of Education), Shanghai Key Laboratory of Psychotic Disorders, Institute of Psychology and Behavioral Science and Brain Science and Technology Research Center, Shanghai Jiao Tong University, Shanghai, China; ^3^Center for Teacher Education Research, Beijing Normal University, Key Research Institute of Humanities and Social Sciences in Universities, Ministry of Education of China, Beijing, China

**Keywords:** adolescents, internet addiction, parental mediation, father–child relationship, mother–child relationship

## Abstract

**Introduction:**

Family factors, such as parental mediation on Internet use and parent-child relationships, have been shown to play a crucial role in preventing adolescents' internet addiction. Previous studies have shown a change in characteristics of online risk during adolescents' development. However, it is still of great interest whether such differences applied in the relationships among parent-child relationships, different types of parental mediation and adolescents' internet addiction level. In this study, we investigated the associations between different types of parental mediators and adolescents' internet addiction level and how the associations were mediated by father-child and mother-child relationships. We further investigated whether mediating effect differs between primary and secondary school children.

**Methods:**

Based on a sample of 3,026 school children aged 9–14 years (*M* = 11.56, *SD* = 0.71; 55.25% primary school adolescents, 44.75% secondary school adolescents), a series of Structural Equation Models were applied to investigate the relationships among internet addiction, parental mediation, and parent-child relationship. In addition, a series of multi-group analysis were applied to detect whether there are differences in these relationships between the primary and secondary school group.

**Results:**

The internet addiction level and intensity of parental mediation was higher among primary school adolescents than secondary school adolescents. Parental active mediation and monitoring on internet use were associated with reduced and increased adolescents' internet addiction. Father-child relationship had stronger partial mediating effects on the relationships between parental mediation and adolescents' internet addiction than the mother-child relationship. The relationships among parental mediation, parent-child relationship and internet addiction were more pronounced among primary school adolescents than secondary school adolescents.

**Discussion:**

The findings suggest that good father-child relationships and adequate parental mediation approach, such as active mediation, may contribute to reduction of internet addiction risk in adolescents, especially in primary school adolescents.

## Introduction

According to the report on the internet use of Chinese children [China Internet Network Information Center (CNNIC)], the number of Chinese children internet users (aged between 6 and 18) had consecutively increased for 2 years and reached 183 million in 2020. Although the internet plays an important role in teenagers' studying, recreation, and social interaction, excessive or unlimited use can lead to internet addiction (IA), which is also termed “problematic internet use” or “pathological internet use (Zhou et al., 2018).” It refers to a maladaptive pattern of internet use in which individuals cannot control themselves and experience problematic outcomes (Niu et al., 2013; Zhang et al., 2013). IA in children might not only decrease their general wellbeing (Yavuz, 2019), impair interpersonal relationships (Karaer and Akdemir, 2019), and lead to academic problems (Demir and Kutlu, 2018) but also might be a significant risk factor for psychological disorders (e.g., anxiety and depression) (Li et al., 2020). As it is of great significance to investigate ways to protect children from internet addiction, the role of parents should never be neglected (Chng et al., 2015). Even though it is widely accepted that parents should mediate their adolescents' internet use and the parent–child relationship is closely connected with adolescents' internet addiction (Li et al., 2014; Bleakley et al., 2016; Jang and Ryu, 2016), the specific approach during childhood development has not yet been deeply discussed. Considering the important role of parents in mediating adolescents' internet use, it is worth further pursuing the relationship between parental mediation, the parent–child relationship, and internet addiction during children's development.

According to parental mediation (PM) theory, parents utilize a variety of direct and indirect strategies in mediating adolescents' online access and consumption (Livingstone and Bober, 2006; Clark, 2011). PM refers to parental management of the relationship between children and media (Livingstone and Helsper, 2008). Previous studies on media use have categorized PM into three dimensions: instructive or active, restrictive, and co-use mediation (Nikken and Jansz, 2006). However, compared to the mediation of media use, the mediation of internet use is more complicated. Given the characteristic of internet use, researchers proposed five types of PM: (1) co-use: Parents are present or sometimes involved when adolescents use the internet; (2) active mediation: Parents guide the adolescents' internet use by discussing the content of internet activities; (3) restrictive mediation: Parents restrict adolescents online activities by setting rules; (4) monitoring: Parents sometimes check or read adolescents' online activity records; (5) technical restrictions: Parents use technology tools to filter or restrict or monitor adolescents' online activities (Livingstone et al., 2011; Sonck et al., 2013; Nikken and Jansz, 2014).

Many studies have found PM, in general, was positively associated with reduced risk of IA (Siomos et al., 2012; Chng et al., 2015; Bleakley et al., 2016). However, the preferred implementation of mediation regarding adolescents' internet use is still under debate (Symons et al., 2017). Some studies argued that parents may have taken excessive responsibility for supervising adolescents' media use, such as internet use (Hasebrink et al., 2009). Nielsen et al. (2019), in a systematic literature review, reported no PM type consistently leads to reduced or increased online problems. For example, mediation was found to exert a positive effect on problematic internet use in the study of Soh et al. (2018) but showed no association with problematic internet use in the study of Chang et al. (2015). Livingstone and Helsper (2008) suggested that the associations between different types of mediation and IA were different. For example, parental restriction, rather than active co-use, was associated with reduced online risks (Livingstone and Helsper, 2008). In a word, it is worth systematically investigating how different types of PM affect adolescents' IA, thus providing reference for parents to choose a better manner in participating in adolescents' internet use.

It has been suggested that the quality of the parent–child relationship (PCR) may mediate the effect of PM on internet use (Su et al., 2018). Previous studies showed that parental mediation is related to the satisfaction of children's psychological need; therefore, insert an effect on the parent–child relationship, which further leads to the risk of internet addiction if children's psychological need is not satisfied (Kwon et al., 2011; Benrazavi et al., 2015; Su et al., 2015). Appropriate PM is beneficial to enhance the parental–child relationship, therefore, lead to decreased online risk (Yang et al., 2021). Excessive PM or intervention behavior may prevent children from their basic psychological demands, such as autonomy, competence, and relatedness, therefore, generating poor relationships (Benrazavi et al., 2015; Su et al., 2015). In this case, children are prone to seek compensate online to satisfy their psychological needs (Ryan and Deci, 2000). However, no study has yet examined how the PCR mediates the effect of different types of PM on IA.

It should also be noted that fathers and mothers may play different roles in adolescents' internet use. Prior studies have mostly focused on how the mother–child relationship or parent–child relationship, in general, may affect adolescents' internet use (Zhu et al., 2015; Nguyen et al., 2022). Recent studies have accumulated evidence specifically focusing on the importance of the father–child relationship in the adolescents' problematic behaviors or online risks (Fosco et al., 2012; Su et al., 2018). For example, Su et al. (2018) reported father–child relationship (FCR), but not mother–child relationship (MCR) played a vital role in adolescents' internet game disorders, pointing to the assumption that differences may exist in the mediation effects of MCR and FCR on the association between PM and IA. Further investigation into such differences may contribute to a deeper understanding of the responsibility of the father and mother of the family in protecting adolescents from internet risk.

Adolescence is commonly regarded as a crucial development phase, which is associated with a change in biological, social, and cognitive domains. Early adolescents are usually referred to as 10–12-year-old adolescents from primary school (4–6 grades). Mid-adolescents are referred to as 13–15-year-old adolescents from secondary school (7–9 grades) (Zou and Wu, 2020). In China, the developmental stage transition from early to mid-adolescence is closely associated with a change in the school context. Prior studies have provided enough evidence of the difference in the characteristics of problematic internet use between early and mid-adolescents (e.g., Gomez-Baya et al., 2017; Liu et al., 2021). For example, early adolescences tend to use internet for their sensation-seeking tendencies (Odgers and Jensen, 2020), while mid-adolescences are characterized by increased socialization demand (Leung, 2007). However, during the transfer from early to mid-adolescents, parents become less likely to engage in mediation behavior (Rosen et al., 2008). Further research on these differences may be critical for parents' implementation of targeted guidance or mediation (Odgers and Jensen, 2020).

In the current study, we aim to investigate the association between different dimensions of PM and adolescents' IA, as well as the role of PCR and adolescents' grades in the associations. Specifically, we aim to investigate the following research questions: (1) whether the different dimensions of PM are associated with adolescents' IA in different ways; (2) whether FCR or MCR account for the relationships between PM and IA level, in another word, whether FCR or MCR mediate the relationships between PM and IA level; (3) whether there are group differences in the relationships investigated in (1) and (2) between adolescents with different grade levels (primary vs. secondary school). Grade level was considered as a variable representing developmental stages (Liu et al., 2020a,b, 2021). We expected (1) different dimensions of PM are differently associated with IA. Reduced IA is associated with more active parental mediation. (2) FCR and MCR play mediation effects on the associations between PM and IA. (3) Group differences exist in the relationships among PM, IA and FCR/MCR. The relationships are stronger among the primary school group than the secondary school group.

## Materials and methods

### Participants

The participants included fourth to ninth graders recruited from primary and secondary schools in Shenzhen, China. A total of 4,114 adolescents were recruited. Only those adolescents with experience in internet use were included in the final sample. Of total, 976 participants were, therefore, excluded for lack of internet use experience. Further attrition was mainly due to invalid responses, including 116 participants who failed to complete the questionnaire. The final sample consisted of 3,026 adolescents. The mean age of the adolescents was 11.56 years (SD = 0.71, ranging from 9 to 14 years). Among these adolescents, 55.25% were enrolled in primary school, 52.91% were men, and 19.71% had no siblings.

### Procedure

Prior to data collection, written informed consent was obtained from the teachers and parents of the participants. Participants were asked to complete a series of online self-report questionnaires under the supervision of their teachers. The questionnaires were designed regarding to their internet use and relationship with their parents. The questionnaire included a question: “Do you have experience in Internet use?” Participants answering “no” were excluded for lack of internet use. All participants were informed to finish the questionnaires independently and honestly. Meanwhile, all participants were informed of the strict confidentiality of their answers and their freedom to quit during the whole procedure. Ethics approval for the whole study was obtained from the Ethics Committee of the Nanjing Normal University of Special Education.

### Measures

#### Internet addiction test

The Internet Addiction Test Scale (IAT, Young, 1998) was used to measure the level of adolescents' IA. This scale was developed for assessment of symptoms of IA and compulsivity in a variate of test settings. The scale was created by adapting the Diagnostic and Statistical Manual of Mental Disorders, Fourth Edition (DSM-IV) criteria for pathological gambling. It has been widely applied in previous studies as a measurement tool of IA (Gentile, 2009; Kwon et al., 2011; Yu et al., 2015; Zhu et al., 2015; Su et al., 2018). Adolescents were asked to answer 20 questions regarding their feelings about using the internet. The 20 items consist of six dimensions for IA symptoms which are: salience (five items, such as *How often do you fear that life without the Internet would be boring, empty, and joyless?*), excessive use (five items, such as *How often do you find that you stay online longer than you intended?*), neglect work (three items, such as *How often does your job performance or productivity suffer because of the Internet?*), anticipation (two items, such as *How often do you find yourself anticipating when you will go online again?*), lack of control (three items, such as *How often do you try to cut down the amount of time you spend online and fail?*), and neglect social life (two items, such as *How often do you form new relationships with fellow online users?*). Items were randomized across dimensions. Items were rated on a 5-point Likert scale (1 = never to 5 = always), with the exception for four inverse-scored items. All items were averaged to create an overall IA level with a higher score corresponding to a higher level of IA. The composite score was obtained by averaging the scores of the 20 items. Reliability and validity of the measurement were good: Cronbach's alpha = 0.94 and KMO = 0.97 (*p*-value < 0.01).

#### Parental mediation of online activities questionnaire

Measurement of PM of adolescents' internet activities was applied with the Parental Mediation of Online Activities Questionnaire (Livingstone et al., 2011; Wu et al., 2019). The measurement tool was originally developed by Livingstone and Helsper (2008) and adapted by Livingstone et al. (2011) in a mass survey on EU kid's internet use (Livingstone et al., 2011, 2014; Smahel et al., 2020) and has shown good reliability and validity in the previous study (Chang et al., 2015). In this study, we used the Chinese version of this questionnaire (Wu et al., 2019) for measurement of PM on adolescents' internet use. Adolescents were asked to compete the questionnaire, including 25 items consisting of five dimensions of PM: monitoring (four items, such as *Does your parents check your email or instant message?*), active mediation (eight items, such as *Does your parents explain to you good and bad websites?*), restrictive mediation (six items, such as *Does your parents restrict you when you download music or movies?*), co-use (three items, such as *Does your parents sit beside you when you are using Internet*), and technical restrictions (four items, such as *Does your parent use software to block spam emails or virus*). Items were rated on a 5-point Likert scale (1 = never to 5 = always) with the exception of five inverse-scored items. Item scores were averaged within each dimension, generating five average scores with a higher score corresponding to higher level of PM. Reliability and validity of the measurement were good: Cronbach's alpha = 0.87 and KMO = 0.91 (*p*-value < 0.01). The Cronbach's alpha values for each dimension are as follows: monitoring: 0.905, active mediation: 0.898, restrictive mediation: 0.829, co-use: 0.637, and technical restriction: 0.714.

#### Parent–child relationship scale

The Parent–Child Relationship Scale was developed by Buchanan et al. (1991) and widely used for assessment of closeness between adolescents and their parents (Zhang et al., 2011; Chen et al., 2022). The scale is composed of 10 items regarding to adolescents' feelings toward their father or mother. In this study, adolescents were asked to answer 20 questions, 10 of them on FCR and 10 on MCR, such as “How openly do you talk with your father/mother?” or “How interested is your mother/father in talking to you when you want to talk?.” Items were rated on a 5-point Likert scale (1 = not at all to 5 = very much). Items were averaged to create separate scores for mothers and fathers, with higher scores indicating a higher level of closeness with their father or mother. Cronbach's alpha was 0.90 for the father subscale and 0.91 for the mother subscale. KMO was 0.93 (*p*-value < 0.01) for the father subscale and 0.94 for the mother subscale (*p*-value < 0.01).

### Statistical approach

First, descriptive statistics for all study variables and correlations were calculated. Second, we applied a series of structural equation models (SEMs) to investigate the relationships among internet addiction (IA), parental mediation (PM), and parent–child relationship (PCR). In addition, a series of multi-group analysis were applied to detect whether there are differences in these relationships between the primary and secondary school group. All statistical analyses were conducted with Mplus Version 7.0 and R 3.6.3. Missing data were handled using the full-information maximum likelihood (FIML) estimation. The bootstrap method was applied to estimate the indirect effects (Bollen and Stein, 1992; Enders, 2002). We defined good model fit with the following criteria: CFI > 0.95, RMSEA < 0.06, and SRMR < 0.08. Given the larger sample size of the current study, χ^2^-test result was not considered as key criteria for model fit because χ^2^ could be particularly sensitive to the large sample size (Hoyle, 2012; Bergh, 2015).

## Results

### Common method bias

The common method bias was examined using Harman's single-factor test (Wu et al., 2022). The results of the exploratory factor analysis showed that the variance interpretation percentage of the first principal component was 24.634%, lower than 40%. This indicates that common method bias had little effect on the overall results of the present study (Ashford and Tsui, 1991).

### Preliminary analyses

Table 1 reported the means, standard deviations, and correlations of the major study variables. Gender (1 for male; 0 for female), grade (1 for primary school; 0 for secondary school), and sibling status (1 for non-only child; 0 for only child) were dummy-coded variables. The results indicate that most of the bivariate correlations of the major variables in our hypothesized models were significant. Adolescents' IA level was negatively correlated with MCR and FCR. The correlations of PM with IA and the parent–child relationship were dependent on the dimension of PM. Parental monitoring was positively correlated with IA level and negatively correlated with MCR and FCR. Restrictive mediation was positively correlated with IA and negatively correlated with MCR. Parent co-use and technical restrictions were positively correlated with the IA level and FCR. Parent active mediation was negatively correlated with IA level, while it was positively correlated with MCR and FCR. Adolescents' grades were positively associated with parental monitoring, restrictive mediation, technical monitoring, and IA level. No significant correlation was found between IA and other demographic variables such as adolescents' gender, age, and sibling status.

**Table 1:** Descriptive statistics and correlations for all variables (*N* = 3,026).

**Variable**	**PM-D1**	**PM-D2**	**PM-D3**	**PM-D4**	**PM-D5**	**MCR**	**FCR**	**IA**
PM-D1	1							
PM-D2	−0.018	1						
PM-D3	0.370^**^	0.118^**^	1					
PM-D4	0.292^**^	0.250^**^	0.389^**^	1				
PM-D5	0.394^**^	0.241^**^	0.483^**^	0.464^**^	1			
MCR	−0.193^**^	0.500^**^	−0.043^*^	0.094^**^	0.017	1		
FCR	−0.172^**^	0.543^**^	−0.002	0.120^**^	0.085^**^	0.594^**^	1	
IA	0.217^**^	−0.201^**^	0.101^**^	0.057^**^	0.072^**^	−0.229^**^	−0.267^**^	1
Gender	−0.032	−0.023	−0.006	0.006	0.025	0.005	0.059^**^	0.035
Age	0.001	−0.002	−0.019	−0.001	0.015	0.018	0.012	0.006
Grade	0.082^**^	0.009	0.112^**^	0.019	0.023^*^	−0.043^*^	−0.028	0.257^**^
Only-child	−0.013	−0.078	−0.055	−0.073	−0.058	−0.059	−0.045	0.002
Mean	2.021	3.511	2.603	2.609	2.538	3.601	3.292	2.112
SD	1.068	0.991	0.966	0.945	0.986	0.773	0.826	0.785

IA, internet addiction; PM-D1 to PM-D5, five dimensions (monitoring, active mediation, restrictive mediation, co-use, and technical restrictions) of parental mediation; MCR, mother–child relationship; FCR, father–child relationship.

^*^p < 0.05;

^**^ p < 0.001.

### Association between different dimensions of parental mediation and internet addiction level

**Figure 1:**
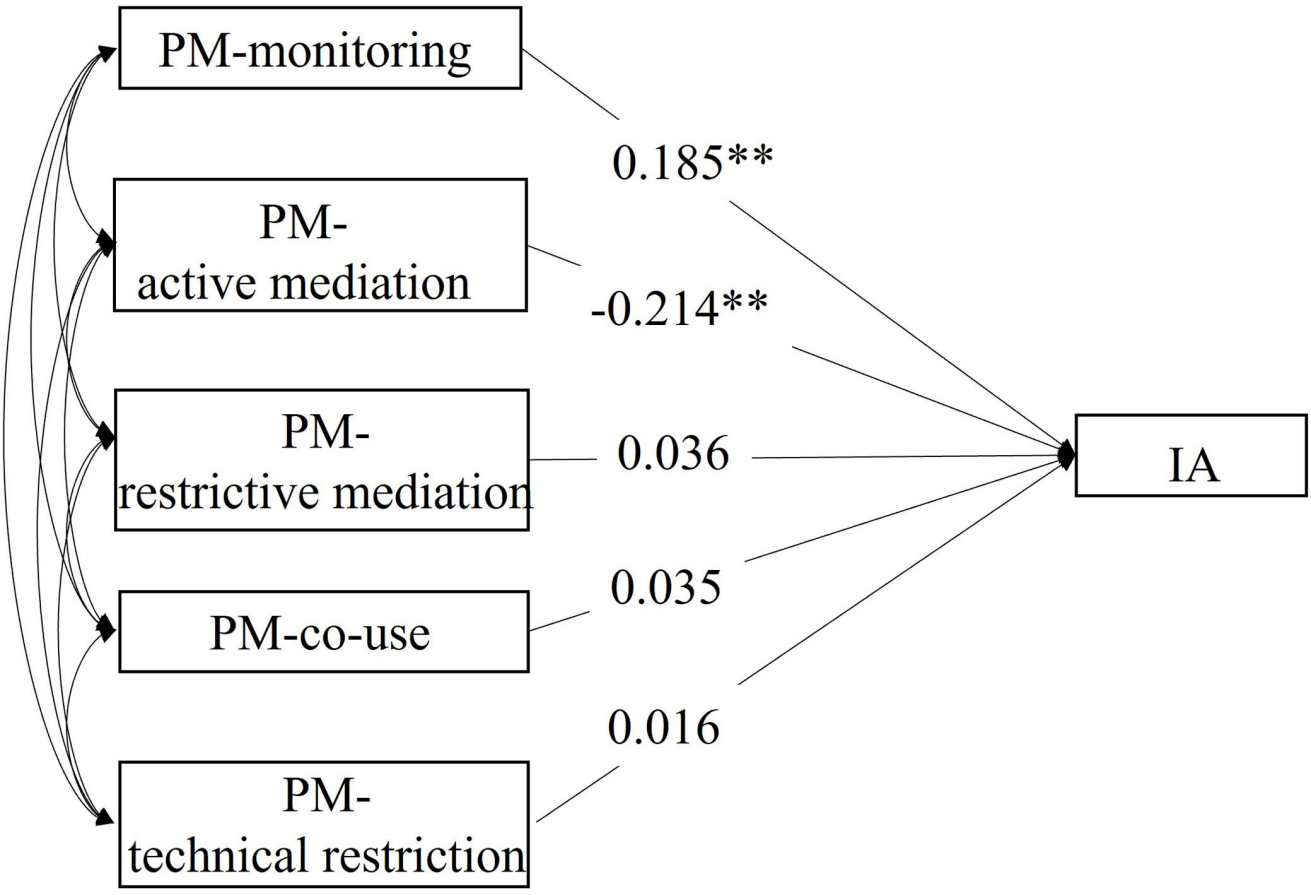
Structural equation model investigating the direct effect of five dimensions of PM on IA level. **p < 0.001.

In order to further investigate the association between different dimensions of PM and IA, model 1 further examined how IA was associated with different dimensions of PM under the SEM framework (Figure 1). Model 1 is a saturated or just-identified model, the parameter estimates of which resulted in perfect model fit values: CFI = 1.000, RMSEA = 0.000, and SRMR = 0.000. Results of model 1 suggested that increased parental monitoring on adolescents' internet use was significantly associated with increased adolescents' IA levels [β: 0.185, *p* < 0.01, CI: (0.153, 0.217)], while active mediation was associated with reduced IA level (β: −0.214, *p* < 0.01, CI: (−0.243, −0.184)]. Restrictive mediation, co-use, and technical restriction were all positively related to IA level, but the regression weights were not significant.

### Mediation effect of PCR on the association between parental mediation and internet addiction

**Figure 2:**
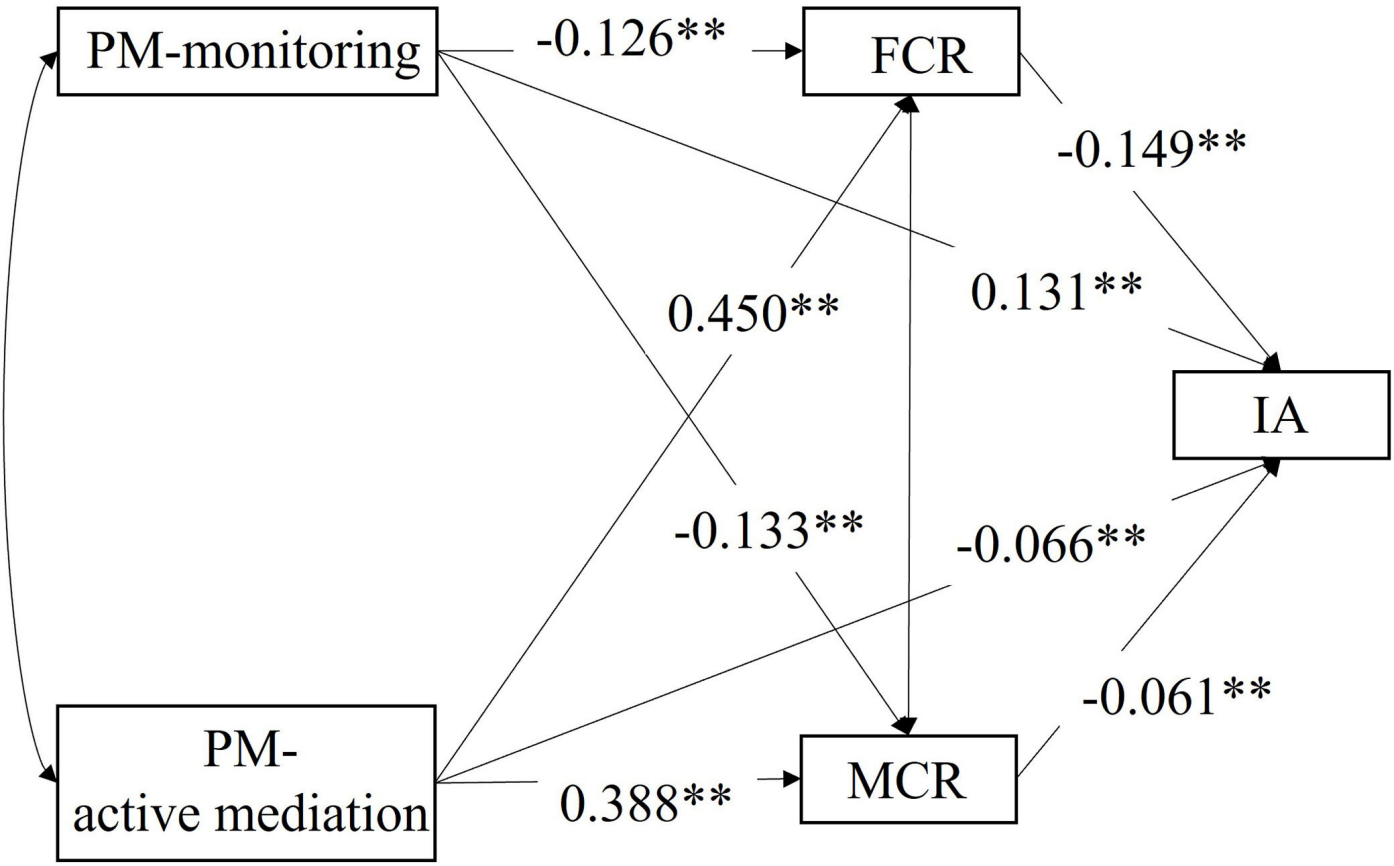
The SEM is investigating how FCR and MCR mediate the effect of PM on IA level. **p < 0.001.

Aligned with a previous study (Su et al., 2018), PCR could be considered as a mediator between the two dimensions of PM (i.e., monitoring and active mediation) and IA. As suggested by model 1, restrictive mediation, co-use, and technical monitoring had no direct effect on the IA level. Therefore, we only considered monitoring and active mediation in the analysis of mediation effects in model 2 (Wen et al., 2004). In model 2, FCR and MCR were added to model 1 as mediators (Figure 2). According to model 2, four mediation paths were assumed: (1) FCR acted as mediator between monitoring and IA; (2) MCR acted as mediator between monitoring and IA; (3) FCR acted as a mediator between active mediation and IA; and (4) FCR acted as a mediator between active mediation and IA. Model 2 had a good fit: CFI = 1.000, RMSEA = 0.000, SRMR = 0.000.

Results of model 2 suggested mediation effect of FCR and MCR: Parental monitoring had a positive direct effect on IA [β = 0.131, *p* < 0.001, 95% CI: (0.109, 0.154)]. IA was negatively associated with FCR [β = −0.149, *p* < 0.001, 95% CI: (−0.187, −0.111)] and MCR [β = −0.061, *p* < 0.001, 95% CI: (−0.100, −0.022)], while parental monitoring was negatively associated with FCR [β = −0.126, *p* < 0.001, 95% CI: (−0.147, −0.104)] and MCR [β = −0.133, *p* < 0.001, 95% CI: (−0.153, −0.114)]. The mediation effects of FCR and MCR on the relationship between parental monitoring and IA were 0.019 [*p* < 0.001, 95% CI: (0.013, 0.024)] and 0.008 [*p* < 0.001, 95% CI: (0.003, 0.013)]. Parent active mediation had a negative direct effect on IA [β = −0.066, *p* < 0.001, 95% CI: (−0.094, −0.038)]. IA was negatively associated with FCR [β = −0.149, *p* < 0.001, 95% CI: (−0.187, −0.111)] and MCR [β = −0.061, *p* < 0.001, 95% CI: (−0.100, −0.022)], but parent active mediation was positively associated with FCR [β = 0.450, *p* < 0.001, 95% CI: (0.425, 0.475)] and MCR [β = 0.388, *p* < 0.001, 95% CI: (0.363, 0.413)]. The mediation effect of FCR and MCR on the relationship between parent active mediation and IA was −0.067 [*p* < 0.001, 95% CI: (−0.084, −0.050)] and −0.024 [*p* < 0.001, 95% CI: (−0.039, −0.009)].

### Multi-group comparison in the associations between parental mediation and internet addiction

**Figure 3:**
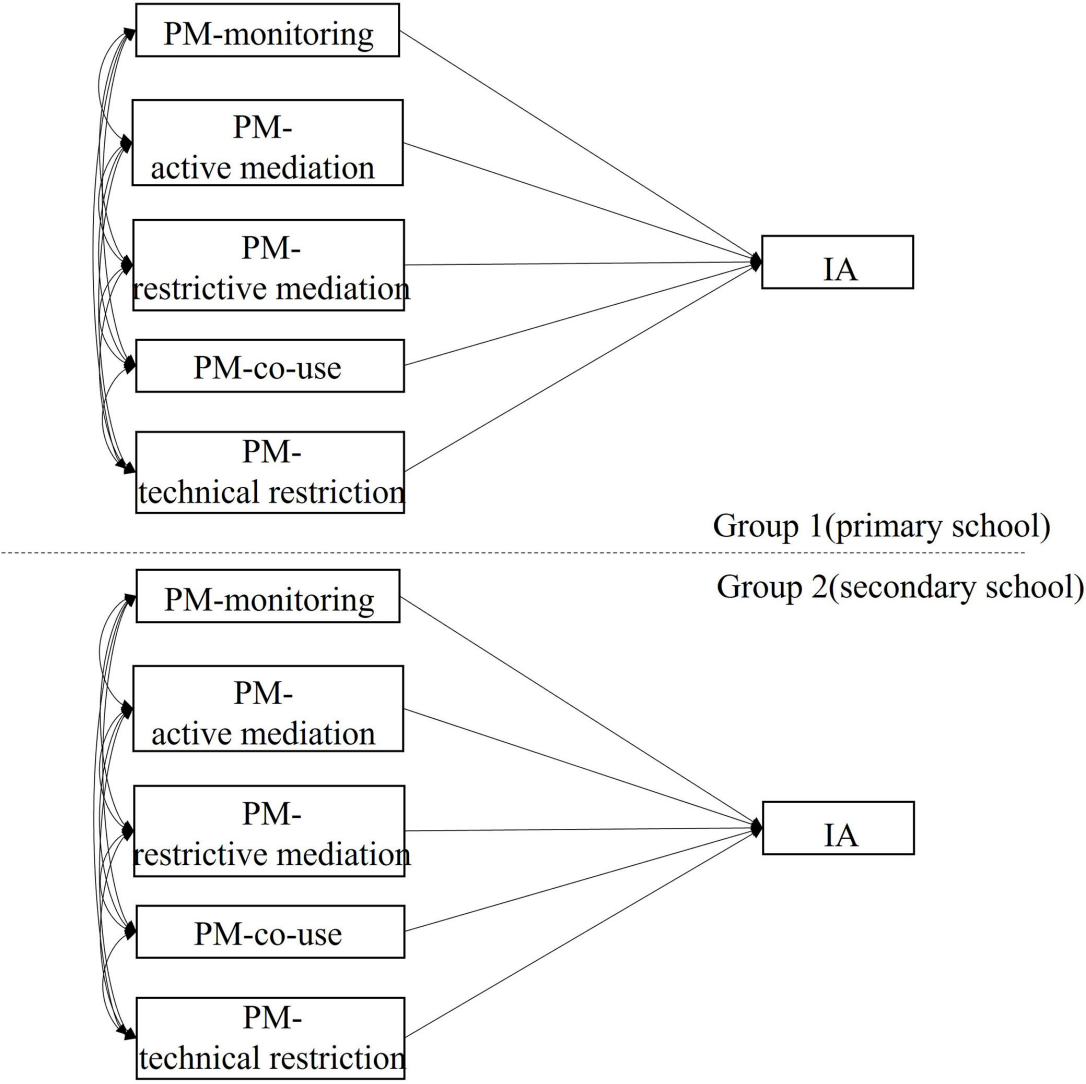
Schematic representation of multi-group comparison model investigating group difference in the effects of PM on IA. The group was set as a categorical variable indicating membership of grade groups.

Differences in the association between PM and IA among the grade groups were examined through multi-group comparisons (model 3). The group membership was dummy coded as 1 = primary school and 0 = mid-adolescents. Equivalence of effects across grades was tested with χ^2^-difference test (i.e., examining the χ^2^-difference test across two models: a constrained model with a specific indirect path, fixed at equal across groups, and an unconstrained model with the specific indirect path, estimated freely across groups). Construction of model 3 is illustrated in Figure 3.

Results, as displayed in Table 2, showed that the effect of active parental mediation on IA level was significantly stronger in the primary school group [effect: −0.231, 95% CI: (−0.269, −0.194)] as compared with the secondary school group [effect: −0.126, 95% CI: (−0.152, −0.100)]. No significant group difference was found in the effects of monitoring, restrictive mediation, co-use, and technical monitoring on the IA level. Furthermore, we found a significant effect of co-use on IA among secondary school students [effect: 0.039, 95% CI: (0.010, 0.068)] and the significant effect of technical monitoring on IA among primary school [effect: 0.042, 95% CI: (0.004, 0.080)] as complementary result of model 1.

**Table 2:** Estimations of effects of PM on IA.

**Parameters**	**All participants** **(95% CI)**	**Primary school group** **(95% CI)**	**Secondary school group** **(95% CI)**	**Wald test *p*-value**
PM-D1 → IA	0.185** [0.153, 0.217]	0.148** [0.114, 0.182]	0.105** [0.079, 0.132]	0.085
PM-D2 → IA	−0.214** [−0.243, −0.184]	−0.231** [−0.269, −0.194]	−0.126** [−0.152, −0.100]	0.001
PM-D3 → IA	0.036 [−0.000, 0.070]	0.006 [−0.033, 0.046]	0.012 [−0.017, 0.042]	0.828
PM-D4 → IA	0.035 [0.002, 0.068]	0.031 [−0.010, 0.072]	0.039* [0.010, 0.068]	0.773
PM-D5 → IA	0.016 [−0.020, 0.052]	0.042* [0.004, 0.080]	−0.004 [−0.036, 0.027]	0.081

IA, internet addiction; PM-D1 to PM-D5, five dimensions (monitoring, active mediation, restrictive mediation, co-use, and technical restrictions) of parental mediation.

*p < 0.05; ** p < 0.001.

### Multi-group comparison in the mediation effects of the father–child relationship and mother–child relationship

**Figure 4:**
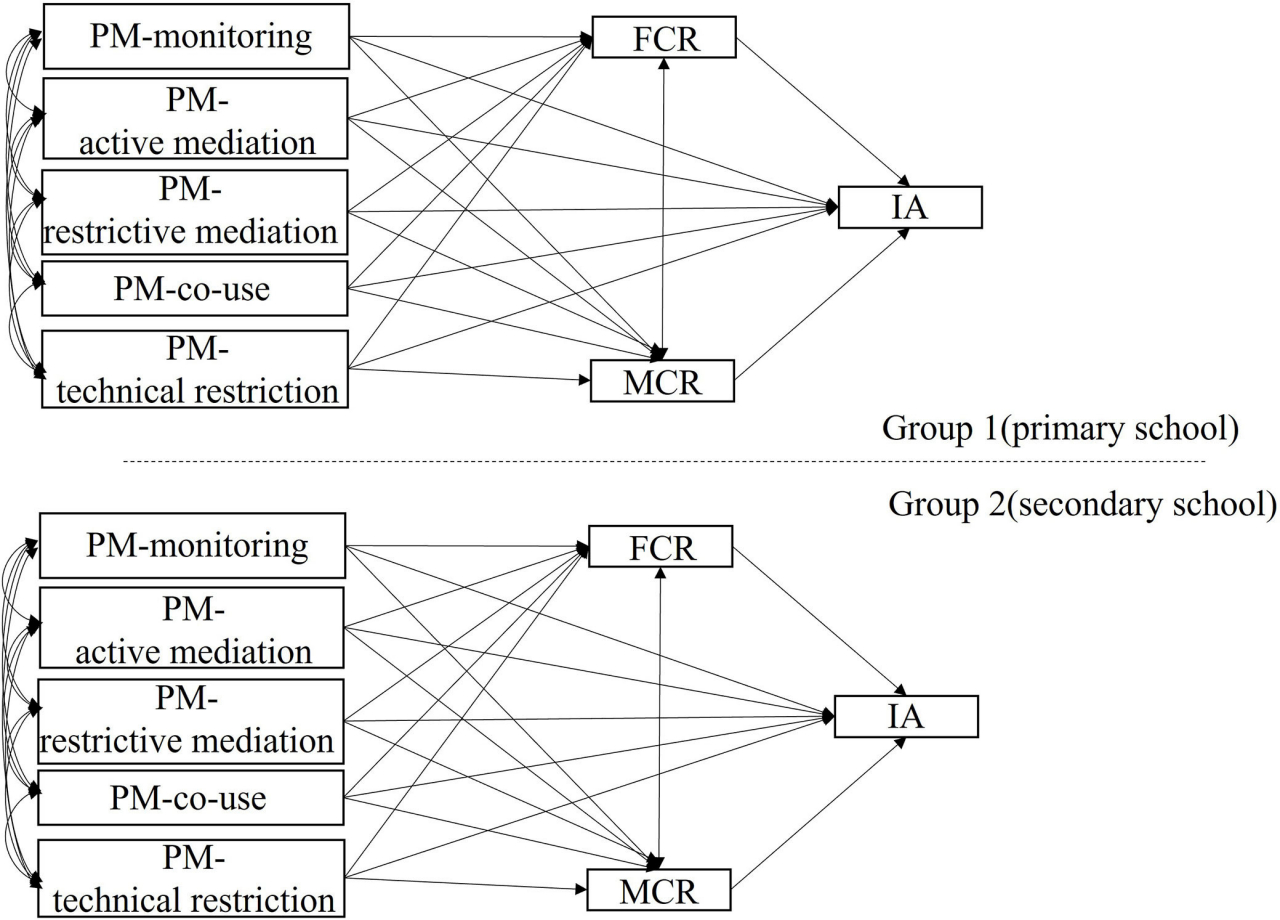
Schematic representation of multi-group SEM investigating group difference in the indirect effects from PM to IA. Group was set as a categorical variable indicating membership of primary or secondary school groups.

Adolescents' grade differences in the mediation model were further examined through multi-group comparisons (model 4, Figure 4). We tested the equivalence of each mediation effect by grade using a χ^2^-difference test. The results show that four mediation effects were significantly different according to an adolescent's grade (Table 3). Specifically, parental monitoring was indirectly associated with IA through stronger FCR in the primary school group [indirect effect =0.030, 95% CI (0.021, 0.039)] than in secondary school [indirect effect = 0.013, 95% CI (0.008, 0.019)]. Parental monitoring was indirectly associated with IA through MCR in the primary school group [indirect effect = 0.016, 95% CI (0.009, 0.024)], but not in secondary school [indirect effect = 0.002, 95% CI (−0.002, 0.006)]. Active parental mediation was indirectly associated with IA through stronger FCR in the primary school group [indirect effect = −0.105, 95% CI (−0.128, −0.082)] than in secondary school [indirect effect = −0.043, 95% CI (−0.059, −0.027)]. Active parental mediation was indirectly associated with IA through MCR in the primary school group [indirect effect = −0.049, 95% CI (−0.069, −0.028)], but not in secondary school [indirect effect = −0.008, 95% CI (−0.022, 0.007)]. Parental co-use was indirectly associated with IA through FCR and MCR in the primary school group [indirect effect *via* FCR: −0.009, 95% CI: (−0.016, −0.001); indirect effect *via* MCR: −0.006, 95% CI: (−0.010, −0.001)], but not in secondary school [indirect effect *via* FCR: −0.003, 95% CI: (−0.007, 0.001); indirect effect *via* MCR: −0.001, 95% CI: (−0.002, 0.001)]. Parental restriction and technical monitoring were not significantly indirectly related to IA *via* FCR or MCR in primary and secondary groups. The difference in the indirect effects between primary and secondary school was non-significant.

**Table 3:** Group differences in estimations of indirect effects from PM to IA.

**Parameters**	**All participants** **(95% CI)**	**Primary school group** **(95% CI)**	**Secondary school group** **(95% CI)**	**Wald test *p*-value**
PM-D1 → FCR	−0.126** [−0.147, −0.104]	−0.126** [−0.151, −0.101]	−0.139** [−0.169, −0.108]	
FCR → IA	−0.149** [−0.187, −0.111]	−0.240** [−0.289, −0.190]	−0.097** [−0.133, −0.062]	
Indirect effect	0.019** [0.013, 0.024]	0.030** [0.021, 0.039]	0.013** [0.008, 0.019]	0.003
PM-D1 → MCR	−0.133** [−0.153, −0.114]	−0.133** [−0.157, −0.108]	−0.111** [−0.140, −0.082]	
MCR → IA	−0.061** [−0.100, −0.022]	−0.121** [−0.173, −0.069]	−0.020 [−0.058, 0.018]	
Indirect effect	0.008* [0.003, 0.013]	0.016** [0.009, 0.024]	0.002 [−0.002, 0.006]	0.003
PM-D2 → FCR	0.450** [0.425, 0.475]	0.439** [0.412, 0.466]	0.442** [0.411, 0.472]	
FCR → IA	−0.149** [−0.187, −0.111]	−0.240** [−0.289, −0.190]	−0.097** [−0.133, −0.062]	
Indirect effect	−0.067** [−0.084, −0.050]	−0.105** [−0.128, −0.082]	−0.043** [−0.059, −0.027]	0.000
PM-D2 → MCR	0.388** [0.363, 0.413]	0.397** [0.371, 0.422]	0.381** [0.353, 0.410]	
MCR → IA	−0.061** [−0.100, −0.022]	−0.123** [−0.174, −0.071]	−0.021 [−0.059, 0.017]	
Indirect effect	−0.024** [−0.039, −0.009]	−0.049** [−0.069, −0.028]	−0.008 [−0.022, 0.007]	0.003
PM-D3 → FCR	–	−0.017 [−0.046, 0.012]	−0.022 [−0.056, 0.012]	
FCR → IA	–	−0.240** [−0.289, −0.190]	−0.097** [−0.133, −0.062]	
indirect effect	–	0.004 [−0.003, 0.011]	0.002 [−0.001, 0.006]	0.657
PM-D3 → MCR	–	−0.022 [−0.052, 0.007]	−0.034** [−0.067, −0.022]	
MCR → IA	–	−0.121** [−0.173, −0.069]	−0.020 [−0.058, 0.018]	
Indirect effect	–	0.003 [−0.001, 0.006]	0.001 [−0.001, 0.002]	0.379
PM-D4 → FCR	–	0.036** [0.006, 0.066]	0.033 [−0.001, 0.067]	
FCR → IA	–	−0.240** [−0.289, −0.190]	−0.097** [−0.133, −0.062]	
Indirect effect	–	−0.009** [−0.016, −0.001]	−0.003 [−0.007, 0.001]	0.224
PM-D4 → MCR	–	0.047** [0.018, 0.076]	0.039** [0.007, 0.072]	
MCR → IA	–	−0.121** [−0.173, −0.069]	−0.020 [−0.058, 0.018]	
Indirect effect	–	−0.006** [−0.010, −0.001]	−0.001 [−0.002, 0.001]	0.062
PM-D5 → FCR	–	0.012 [−0.017, 0.040]	0.016 [−0.019, 0.052]	
FCR → IA	–	−0.240** [−0.289, −0.190]	−0.097** [−0.133, −0.062]	
Indirect effect	–	−0.003 [−0.010, 0.004]	−0.002 [−0.005, 0.002]	0.763
PM-D5 → MCR	–	−0.027 [−0.055, 0.001]	−0.043** [−0.077, −0.009]	
MCR → IA	–	−0.121** [−0.173, −0.069]	−0.020 [−0.058, 0.018]	
Indirect effect	–	0.003 [−0.001, 0.007]	0.001 [−0.001, 0.003]	0.292

IA, internet addiction; PM-D1 to PM-D5, five dimensions (monitoring, active mediation, restrictive mediation, co-use, and technical restrictions) of parental mediation; MCR, mother–child relationship; FCR, father–child relationship.

*p < 0.05; ** p < 0.001.

## Discussion

The current study investigated how different aspects of PM on adolescents' internet use may affect adolescents' IA level. We also investigated the role of the parent–child relationship in the above relationship, as well as the grade difference. Main results of the current study are as follows: (1) Reduced IA level was associated with increased parent active mediation and reduced parental monitoring. (2) Both FCR and MCR mediated the relationship between monitoring and IA level, as well as the relationship between active mediation and IA level. FCR had a stronger mediation effect than MCR. (3) The association between PM and IA differed between early and mid-adolescences. Specifically, active mediation had a significantly stronger effect on IA in primary school adolescences compared with the secondary school group. (4) The mediation effects of FCR and MCR differed between early and mid-adolescences: Both FCR and MCR had a stronger mediation effect for the primary school group as compared with the secondary school group. For secondary school group, MCR mediated only the relationship between parental monitoring and IA, but not the relationship between active mediation and IA.

### The effect of parental mediation on internet addiction

Results of the current study indicated that different facets of PM predict adolescents' IA level in different directions. Parental monitoring positively predicted IA level and active mediation negatively predicted IA level. Consistent with previous studies (e.g., Lwin et al., 2008; Youn, 2008), active parental mediation played a positive role in protecting adolescents from online risks. Active mediation behavior from a parent, such as communication and proper help, may provide adolescents with psychological support, therefore reducing the risk of IA (Zhang et al., 2019).

However, the result that parental monitoring positively predicted IA level was controversial with some previous findings (e.g., Lin et al., 2009; Chang et al., 2015; Ding et al., 2017) that parental monitoring inhibits adolescents' IA level. As pointed out by Hu and Wang (2022) in a study on adolescents' problematic mobile phone usage, the effect of parental monitoring on their addictive behavior interacts with adolescents' self-control level. Therefore, we suggest that the relationship between parental monitoring and adolescents' IA level should be further compared between adolescents with low- and high-self-control. Moreover, given that Chinese parents tend to emphasize parental authority during monitoring (Chao and Tseng, 2002), we suggest that under the Chinese culture, a high level of parental monitoring might lead to adolescents' negative emotion, such as stress or reverse psychology (Chong et al., 2014; Ma et al., 2021), thus increase the risk of getting addicted. Therefore, active mediation, rather than the high intensity of monitoring from parents, might be a preferred approach in protecting adolescents from IA.

It should also be noted that the inconsistency in the association between mediation (such as monitoring) and IA level exists because of discrepancy in the perception of the concepts related to “mediation.” For example, in our study, “monitoring” was referred to as parent's behavior of checking or supervising adolescents' online activities, while in some other studies, “monitoring” was conceptualized as parent's knowledge of adolescents' online activities (e.g., Soh et al., 2018). Such knowledge may be obtained by not only parent's supervision but also adolescents' initiative to talk with the parents. Future studies should pay attention to the uniqueness of conceptualization and measurement tools, therefore inducing consistent clear advice for parents' mediation behaviors.

### The mediation role of father–child and mother–child relationships

As indicated in the results, both FCR and MCR partially mediated the relationships between parental monitoring and IA. We separately discuss the mediation effect on the two dimensions (D1: monitoring; D2: active mediation) investigated in this study, as the directions of effects were differed. According to the results regarding to D1, a high level of parental monitoring leads to poor parent–child relationships and is thus associated with increased IA levels. According to the results regarding to D2, a high level of active parental mediation leads to better parent–child relationships and is thus associated with reduced IA level. These results further suggested that rigorous parental monitoring may turn in poor parent–child relationships, bad social skills, and higher social anxiety (Liu and Kuo, 2007) and, therefore, likely to induce problematic behavior, while active mediation may well-satisfy adolescents' autonomy development requirement *via* communication or support from parents. Parents should consider active mediation rather than monitoring on adolescents' internet use, so as to benefit the parent–child relationship and reduce adolescents' risk of getting addicted.

As compared with MCR, FCR played a more important role in mediating the effect of PM on IA. Previous studies have reported similar results: FCR predicted internet gaming disorder while MCR did not (Liu et al., 2013; Su et al., 2018). Su et al. (2018) suggested that compared with mothers, fathers are more influential in adolescents' behavior rather than emotion (Pinquart, 2017). Similar studies also suggested that mothers take the main responsibility for adolescents' emotional support, rules setting, and organizing their children (Kellerman and Katz, 1978), while the father are more involved in instrumental function, such as physical play (Lamb et al., 1985; Finley and Schwartz, 2006). Based on these results, fathers are more likely to influence adolescents' internet use, as internet plays an important role for instrumental purpose in adolescents' daily life. Moreover, we further argue that as the mother and child have generally higher closeness than the father and a child (Russell and Russell, 1987), the mother–child relationship is less likely to be influenced by an external cause, such as parent's mediation behavior on adolescents' internet use, and therefore less likely to play a mediation role.

### Differences between primary and secondary school groups

Descriptive statistics indicate that primary school adolescents are faced with a higher level of IA. The higher level of IA in primary school adolescents may partly be attributed to relatively low self-control among early adolescents as compared with mid-adolescents and relatively low academic anticipation from primary school on them (Eccles and Roeser, 2011). Accordingly, PM decreases as adolescents grow up. Studies have suggested that decreased PM may result from adolescents' declining acceptance of PM (Livingstone and Bober, 2004; Liau et al., 2008). Thus, older adolescents' increased demand for self-jurisdiction may further explain such transfer.

Multi-group comparison results showed that active mediation had a significantly stronger effect on IA among primary school adolescents. Meanwhile, results showed different mediation effects of parent–child relationships between primary and secondary school groups. Both FCR and MCR played a stronger mediation role in the relationship between IA level and monitoring, as well as active mediation. Such differences could be explained by both adolescents' developmental stage and school setting factors. On the one hand, primary school children at the early adolescence stage are dominantly dependent on parents, while secondary schools' adolescents at mid-adolescence enter a new social–psychological phase of life and become increasingly rely on peers for intimacy and support (Levitt et al., 1993). It is natural that parents' mediation behavior has a stronger effect on early adolescents, and parent–child relationships are more closely related to parent's behavior at adolescents' early adolescent stage. On the other hand, compared with primary school adolescents, Chinese secondary school children endure higher academic stress (Zhao et al., 2015), which leads to decreased interest in social issues (Zhao et al., 2012). Therefore, they are more likely to get alienated from their parents. Their relationships with their parents are less likely to play a role in mediating their internet use.

### Limitations

There are several limitations in the current study. First, the study sample was collected only from primary and secondary schools in Shenzhen, which is one of the most developed metropolises in China. The education quality, school settings, and adolescents' accessibility to internet may differ from those adolescents from less developed regions. Due to the lack of heterogeneity in the study sample, the generalizability of the findings of this study may be affected. Further studies should consider recruiting participants by stratified sampling and recruit participants from multiple areas. Second, the current study was designed as cross-sectional rather than longitudinal, therefore, only able to induce association rather than causal effects between variables. Third, all measures in the current study were completed by adolescents through self-report questionnaires, so there were shared variances between all measures due to single method and informant. Further studies should consider collecting both self-report and parent reports, so as to reduce bias caused by common variance.

## Conclusion

This research found that parent's monitoring and active mediation were independently positively and negatively associated with IA. As compared with MCR, FCR played a stronger mediation role in the above association. Moreover, grade differences existed in the above associations. As compared with mid-adolescences from secondary school, early adolescences were found to have stronger associations in the above relationships. As suggested by results, supportive behaviors, such as substantial communication during adolescents' internet use, are particularly helpful among adolescents, especially early adolescences. Moreover, the role of the father should be especially extended in protecting adolescents from IA risk.

## Data availability statement

The raw data supporting the conclusions of this article will be made available by the authors, without undue reservation.

## Ethics statement

The studies involving human participants were reviewed and approved by Ethics Committee of Nanjing Normal University of Special Education. Written informed consent to participate in this study was provided by the participants' legal guardian/next of kin.

## Author contributions

XL, YD, and LL conceived and designed the research. XL and YD performed the research. XL analyzed the data. XL, YD, XB, and LL contributed to the writing of the manuscript. All authors contributed to the article and approved the submitted version.

## Funding

This research was funded by the Key Project of the National Social Science Fund of China (20ATJ007) to XB, the Youth Foundation for the Natural Science Foundation of Jiangsu province China (BK20191024), and the Natural Science Foundation of the Higher Education Institutions of Jiangsu Province, China (19KJB190003) to YD.

## Conflict of interest

The authors declare that the research was conducted in the absence of any commercial or financial relationships that could be construed as a potential conflict of interest.

## Publisher's note

All claims expressed in this article are solely those of the authors and do not necessarily represent those of their affiliated organizations, or those of the publisher, the editors and the reviewers. Any product that may be evaluated in this article, or claim that may be made by its manufacturer, is not guaranteed or endorsed by the publisher.
